# Inactivation of *SLIT2-ROBO1/2* Pathway in Premalignant Lesions of Uterine Cervix: Clinical and Prognostic Significances

**DOI:** 10.1371/journal.pone.0038342

**Published:** 2012-06-13

**Authors:** Sraboni Mitra, Dipanjana Mazumder-Indra, Ranajit K. Mondal, Partha S. Basu, Anup Roy, Susanta Roychoudhury, Chinmay K. Panda

**Affiliations:** 1 Department of Oncogene Regulation, Chittaranjan National Cancer Institute, Kolkata, West Bengal, India; 2 Department of Gynaecology Oncology, Chittaranjan National Cancer Institute, Kolkata, West Bengal, India; 3 North Bengal Medical College, Siliguri, West Bengal, India; 4 Molecular and Human Genetics Division, Indian Institute of Chemical Biology, Kolkata, West Bengal, India; University of Navarra, Spain

## Abstract

The *SLIT2-ROBO1/2* pathways control diverse biological processes, including growth regulation. To understand the role of *SLIT2* and *ROBO1/2* in cervical carcinogenesis, firstly their RNA expression profiles were screened in 21 primary uterine cervical carcinoma (CACX) samples and two CACX cell lines. Highly reduced expressions of these genes were evident. Concomitant alterations [deletion/methylation] of the genes were then analyzed in 23 cervical intraepithelial neoplasia (CIN) and 110 CACX samples. In CIN, *SLIT2* was deleted in 22% samples compared to 9% for *ROBO1* and none for *ROBO2*, whereas comparable methylation was observed for both *SLIT2* (30%) and *ROBO1* (22%) followed by *ROBO2* (9%). In CACX, alteration of the genes were in the following order: **Deletion:** *ROBO1* (48%) > *SLIT2* (35%) > *ROBO2* (33%), **Methylation:** *SLIT2* (34%) > *ROBO1* (29%) > *ROBO2* (26%). Overall alterations of *SLIT2* and/or *ROBO1* (44%) and *SLIT2* and/or *ROBO2* (39%) were high in CIN followed by significant increase in stage I/II tumors, suggesting deregulation of these interactions in premalignant lesions and early invasive tumors. Immunohistochemical analysis of SLIT2 and ROBO1/2 in CACX also showed reduced expression concordant with molecular alterations. Alteration of all these genes predicted poor patient outcome. Multiparous (≥5) women with altered *SLIT2* and *ROBO1* along with advanced tumor stage (III/IV) and early sexual debut (<19 years) had worst prognosis. Our data suggests the importance of abrogation of *SLIT2-ROBO1* and *SLIT2-ROBO2* interactions in the initiation and progression of CACX and also for early diagnosis and prognosis of the disease.

## Introduction

On worldwide basis, uterine cervical carcinoma (CACX) is the second common gynecological cancer, mostly afflicting the developing nations like India [1]–[3]. Among Indian women CACX ranks second, next only to breast cancer [4]. High-risk Human Papilloma Virus (HPV) is an important etiological agent associated with CACX [5]. It is transmitted sexually and is consequently ubiquitous among sexually active women. Therefore determinants of sexual activity namely; parity, age at sexual debut etc significantly predicts the risk to CACX [6]. Existing evidences indicated an intricate interplay between HPV infection and genetic alterations that critically contributes towards cervical carcinogenesis [7].

Different chromosomal abnormalities namely; deletion, amplification, rearrangement etc involving several chromosomal regions were reportedly associated with the development of CACX [7]–[11]. Our previous study reported frequent deletion (35–38%) and methylation (52%) of *SLIT2*, located at chr. 4p15.31, in CACX of Indian patients [12]. Whereas, predominant hypermethylation (64%) and infrequent deletion (9%) of the same was reported in CACX of Western patients [13]. In normal non-neuronal cells, SLIT2 is a secreted tumor suppressor protein that through binding of its cognate receptors ROBO1/2 and via inhibition of WNT, SDF1 and HGF signaling regulates multiple cellular processes namely; cell cycle, apoptosis, cell-cell adhesion, cellular motility and invasion etc [14]. Interaction between SLIT2 and ROBO1/2 causes activation of srGAP molecules and consequent inactivation of CDC42 (by hydrolysis of bound GTP), leading to cell cycle arrest at G1-S transition [14]. Similarly, SLIT2-ROBO1/2 interaction relieves the inhibition on DCC receptors by ligand Netrin-1, leading to activation of apoptosis via caspases-3 and 9 [14]. In the event of abrogation of SLIT2 and ROBO1/2 interactions the cellular surveillance posited in the form of G1-S checkpoint and apoptosis gets aberrant, giving rise to mutated forms that has the property of conferring greater growth advantage to the cells. This ultimately results in uncontrolled cellular proliferation and promotion of tumorigenesis.

Alike *SLIT2*, frequent methylation (46%) and nominal deletion (10%) frequencies of *ROBO1* was reported in CACX of Western patients [13]. To the best of our knowledge, alterations of *ROBO1* have not been studied in CACX of Indian patients. In addition alterations of *ROBO2* localized 1.3 Mb telomeric to *ROBO1* at chr 3p12.3 region, have not been studied in details in CACX, though its alterations have already been reported in head & neck squamous cell carcinoma [HNSCC] [15].

Intron2 of *ROBO1* harbors two non-coding RNAs [ncRNAs; *BC017743* and *BC043430*], suggesting their probable role in modulating the expression of *ROBO1*. The expression pattern of these ncRNAs in CACX has not yet been studied but their differential expression has been detected in various carcinomas like; lung, breast, oral etc [15], [16].

Therefore, to understand the importance of *SLIT2*-*ROBO1/2* pathway in the development of CACX, it is pertinent to analyze the alterations of these genes in the same set of samples. Thus our study has been focused on the following aspects: (i) quantitative mRNA expression analysis, (ii) alteration (deletion/methylation) analysis and (iii) correlation of molecular alterations of the genes with clinico-pathological parameters (stage, nodes at pathology, HPV status, parity etc) and patient outcome. Our data revealed reduced expression and frequent alterations (37–55%) of *SLIT2* and *ROBO1/2* in primary cervical lesions. In addition, 84% and 80.5% samples showed co-alterations of *SLIT2-ROBO1* and *SLIT2-ROBO2* pairs respectively, indicating the importance of these ligand-receptor interactions in cervical carcinogenesis. Moreover Cox multivariate analysis revealed alterations of *SLIT2* and *ROBO1*, in combination with advanced tumor stage (III/IV), multiparity (≥5) and early sexual debut (<19 years) as determinants of worse prognosis.

## Materials and Methods

### Ethics Statement

The Institutional Ethical board of Chittaranjan National Cancer Institute, Kolkata approved the usage of Human specimens in this study. The above board approved usage of these human clinical samples specifically in this study, pertaining to the involvement of SLIT2-ROBO1/2 signaling in CACX. The tumor specimens were collected from the hospital section of Chittaranjan National Cancer Institute, Kolkata, after obtaining written, informed consent of the concerned patients, in stipulated format, approved by the above mentioned Institutional Ethical board of Chittaranjan National Cancer Institute, Kolkata, India.

### Clinical Specimens and Cell lines

This study included a total of 133 primary cervical lesions and corresponding peripheral blood lymphocytes (PBL) collected from the hospital section of Chittaranjan National Cancer Institute, Kolkata, after institutional consent. The tumors were graded or staged according to FIGO classification. Among these samples, 23 were premalignant/CIN lesions (10 low grade CINI and 13 high grade CINII/III), 56 stage I/II tumors and 54 stageIII/IV tumors (Table 1). The normal cervical tissues (n = 8) were collected from patients with clinically normal cervix, but underwent hystectomy due to other gynecological reasons. These served as controls for RNA/protein study. The tissues (normal/tumors) were either frozen to −80°C or taken in TRIzol reagent (Invitrogen, USA) for RNA isolation or fixed in formalin and paraffin embedded for immuno-histochemistry (IHC). Clinical/follow-up data of the patients were collected from the hospital records. Demographic details were obtained by personally questioning the patients.

**Table 1:** Clinico-pathological features of cervical lesions.

Clinical features	No of Patients	Mean age (yrs)	HPV Positivity					P-value
			HPV +		HPV -	% positivity		
**Tumor stage** §								
CIN	23	36	20	3			87	0.9962
Stage-I	33	47	28	5			85	
Stage-II	27	46	24	3			89	
Stage-III	43	47	37	6			86	
Stage-IV	7	50	6	1			86	
**Tumor differentiation**								
Dysplasia	23	36	20	3			87	0.9491
Well	15	48	13	2			87	
Moderate	80	45	69	11			86	
Poor	15	44	13	2			87	
**Lymph node**								
Node+	32	45	28	4			87.5	0.8450
Node-	101	46	87	14			86	
**Parity (Live births only)**								
Low (0–4)	80	48	68	12			85	0.5451
High (≥5)	53	47	47	6			89	
**Age at sexual debut**								
Early (12–19 y)	73	42	62	11			85	0.5696
Late (>19 y)	60	45	53	7			88	

§ According to The international federation of gynecology and obstetrics (FIGO) classification.

The CACX cell lines: SiHa and HeLa were purchased from National Centre for Cell Sciences, Pune, India and were grown according to supplier’s instructions.

### RNA Expression Analysis

Expression of ROBO1/2, SLIT2 RNAs and the ncRNAs were analyzed by Q-RT PCR in normal cervical tissue (n = 8), CACX samples (#T1–#T21) and cell lines: SiHa and HeLa, using primers given in Table S1. RNA isolation and cDNA preparation were done as outlined by Mitra et al (2010) [17] and Information S1. The real time quantitation of RNA expression was done by Power SYBR-green PCR assay (Applied Biosystems, USA) using ddCt method [18], [19]. The β2-microglobulin was used as the internal control. Details of this method have been included in Information S1.

### 5-aza-dC Treatment of SiHa and HeLa

HeLa and SiHa cell lines were grown in presence and absence of 5-Aza-2′-deoxycytidine (5-aza-dC) for 3–5 days at 5 µM, 10 µM and 20 µM concentrations. The cells were harvested followed by RNA isolation, cDNA preparation and real time quantitations using Power SYBR-green PCR assay according to protocols described above [18], [19].

### Microdissection and DNA Extraction

The clinical specimens comprising mostly of biopsy/surgical materials, were frozen, sectioned in cryomicrotome (5 µm), stained with hematoxylin-eosin and then the contaminant normal cells were removed by microdissection [20] under dissecting microscope (Leica MZ 16, Germany). Microdissected samples containing >60% dysplastic epithelium/tumor cells were taken for DNA isolation according to the standard procedure [20], [21]. Details in Information S1.

### Promoter Methylation Analysis

Methylation sensitive restriction analysis (MSRA) was performed to screen promoter methylation status of *SLIT2* and *ROBO1/2* in a cohort of 23 CIN and 110 CACX samples using primers listed in Table S1 [22]. Among these samples, 17 CIN and 41 CACX samples have already been analyzed for *SLIT2* methylation by a previous study of our laboratory [12]. The methylation sensitive *Hpa*II and its methylation insensitive isochizomer *Msp*I have been used in the analysis. The β-3A adaptin gene (*K1*) was used as digestion control and RARβ2 (*K2*) served as the control for DNA integrity [22].

Methylation analysis was validated in 20 (2 CIN and 18 CACX) randomly selected samples by methylation-specific-PCR (MSP) after bisulphite modification of DNA using primers listed in Table S1B. The genomic DNA (5 µg) was subjected to bisulphite modification followed by PCR amplification of the modified DNA using primers for non-methylation (U) or methylation (M) specific alleles [23]. Details of MSRA/MSP protocols have been included in Information S1.

### Deletion Analysis

Deletion of the *ROBO1/2* and *SLIT2* loci were done in 23 CIN and 110 CACX samples, using microsatellite and exonic markers [12], [15]. Among these, 17 CIN and 41 CACX samples were previously analyzed only (not *ROBO1/2*) for *SLIT2* deletion [12]. In total, 6 microsatellite and 2 exonic markers [(Ensembl release 49; Genome Database); Details in Table S1] were chosen. Among them D3S3507, D3S1274 and D4S1546 were informative (high polymorphic) and the rest were non-informative. The details of the deletion analysis have been included in Information S1. Scoring of loss of heterozygosity [LOH]/deletion and microsatellite size alteration [MA] for informative/non informative markers were done according to standard protocols [12], [20], [24] Details in Information S1.

### Immunohistochemical and Immunocytochemical Analysis

Protein expression of *ROBO1/2* and *SLIT2* was studied by immunohistochemistry (IHC) in normal cervical tissue (n = 8) and primary CACX (n = 15) using primary antibodies [Goat polyclonal IgG sc-16611, sc-16615 and sc-16619 for ROBO1, ROBO2 and SLIT2 respectively] and HRP-conjugated rabbit anti-goat secondary antibody (sc-2768) from Santa Cruz Biotechnology, CA, USA, following standard protocols (Information S1). The scoring of staining frequency and intensity was done according to Perrone et al [2006] [25]. The expression of these proteins in SiHa and HeLa cells were studied by immunocytochemistry. The cells were cultured on coverslips till sub-confluency, fixed with methanol, blocked with BSA, incubated with respective primary antibodies (as above) followed by fluorescein isothiocyanate (FITC)-conjugated secondary antibody (sc-2777), washed and briefly incubated with DAPI (sc-3598) and subsequently mounted on slides, viewed and photographed by fluorescence microscope (Leica DM 4000B; Germany) [17].

### Detection of HPV-16 and HPV-18

Presence of HPV in the cervical lesions were detected by PCR using primers (MY09 and MY11) from the consensus L1 region followed by typing of HPV 16/18, frequent oncogenic variants, in the L1 positive samples [26].

### Statistical Analysis

The χ^2^ analysis determined the association of genetic profile of tumors (alteration of ROBO1/2, SLIT2) with different clinico-pathological parameters. Survival curves were obtained according to Kaplan–Meier method. Cox proportional hazards regression model predicted the significant determinants (genetic/epidemiological) of patient’s survival. Overall survival (OS) was measured from the date of surgery to the date of most recent follow-up or death (upto 5 years). Consistent follow-up records were available for 86 CACX patients with mean and median follow-up of 14±10 months and 10 months respectively. Only these patients were included in the Cox and Kaplan–Meier survival analyses. Probability value (P-value) ≤0.05 was considered statistically significant. SPSS was used to perform all the statistical analyses (SPSS Inc. Chicago, IL, USA).

## Results

### Expression Pattern of *SLIT2* and *ROBO1/2* in CACX

**Figure 1:**
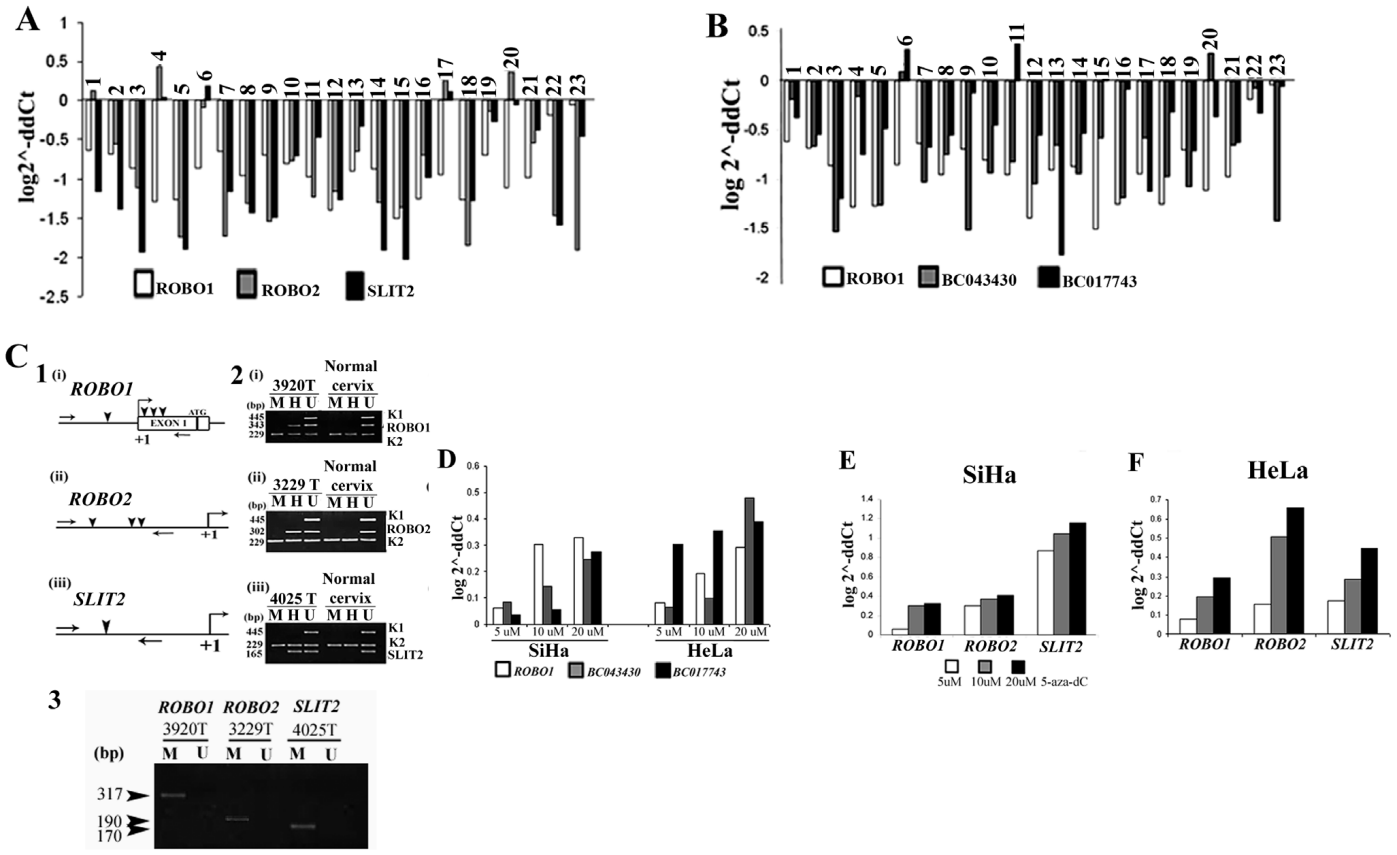
Comparative representation of RNA expression of (A) ROBO1, ROBO2 & SLIT2 and (B) ROBO1, BC017743 & BC043430 in CACX. Bars represented the gene expression normalized to β2- microglobulin and relative to a pool of normal cervical tissues, using the 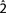-ddCt method. Bars 22 and 23 represent the SiHa and HeLa cell lines, respectively. Analysis of promoter methylation of *ROBO1/2* and *SLIT2* by MSRA and validation by MSP. (**C1**) Schematic representation of promoter regions of candidate genes revealing distribution of *Hpa*II/*Msp*I (CCGG: arrowhead) restriction sites. →: location of methylation primers. **+1**: transcription start site. (**C2**) Representative tumor samples (#3920T, #3229T, #4025T) showing methylated status at different genes, normal cervical tissue were unmethylated. M and H**:** *Msp*I and *Hpa*II digested DNA respectively. U: Undigested DNA. *K1* and *K2*: Controls for DNA digestion and integrity respectively, T: Tumor DNA, N: DNA from corresponding normal tissue. (**C3**) Representative tumor samples showing methylation status of candidate genes by MSP. U: amplicon obtained with primer for bisulphite modified unmethylated DNA, M: amplicon obtained with primer for bisulphite modified methylated DNA, T: Tumor DNA, N: DNA from corresponding normal tissue. (**D**) Reactivation of RNA expression of *ROBO1* and the ncRNAs in SiHa and HeLa cells. Reactivated RNA expression of the ligand-receptor genes in SiHa (**E**) and HeLa (**F**) cell lines in presence of 5 µM, 10 µM and 20 µM 5-aza dC. The bars represent increased gene expression in 5-aza dC treated cells compared to untreated control.

In quantitative RT-PCR analysis, fold reduction in expression of the genes in CACX were in the following order: *SLIT2* (8.8±5.2) > *ROBO1* (8±2.3) > *ROBO2* (7.4±5.6). About 48–57% tumors showed reduction greater than the mean value of expression of the respective genes (Figure 1A,B). Similar trend (except for ROBO1) was observed for the CACX cell lines SiHa and HeLa, in the following order: **SiHa:** SLIT2 (38 folds) > ROBO2 (29 folds), **HeLa:** ROBO2 (80 folds) > SLIT2 (2.8 folds) (Figure 1A,B). Thus, transcriptions of *SLIT2* and *ROBO1/2* were severely impaired during cervical carcinogenesis.

Similarly the expressions of the two ncRNAs were also reduced in both primary CACX and cell lines in the following order: BC043430 (5.8±3.14) > BC017743 (3±2.9) (Figure 1A,B). The ncRNA BC017743 showed 2 folds reduction in SiHa and BC043430 showed 26 folds reduction in HeLa, though ROBO1 was not reduced in any of the cell lines. A comparison of expression levels (ddCt) of the ncRNAs versus *ROBO1*, showed frequent, relatively more reduced expression of *ROBO1* (data not shown), in the primary tumors. These facts probably suggest that the ncRNAs and *ROBO1* were transcribed from separate promoters.

### Frequent Promoter Methylation of *SLIT2-ROBO1/2* in CIN and CACX

To understand the mechanism of impaired expression of *SLIT2* and *ROBO1/2* in cervical lesions, the promoter methylation status of these genes were analyzed. In CIN, methylation frequencies of *SLIT2* (30%, 7/23) and *ROBO1* (22%, 5/23) were high, followed by *ROBO2* (9%, 2/23) (Figure 1C, Table S2A,C). In CACX, the methylation frequencies of *SLIT2* (34%, 37/110) and *ROBO1* (29%, 32/110) were comparable to CIN, though there was substantial increase in methylation frequency of *ROBO2* (26%, 29/110) (Figure 1C, Table S2B,D). In SiHa and HeLa cells, *ROBO2* was methylated, whereas *SLIT2* was methylated only in SiHa. Methylation of *ROBO2* in CACX was not reported earlier. The promoter methylation statuses of the ligand-receptor genes were confirmed in 20 (2 CIN and 18 CACX) randomly selected samples by MSP and the results were concordant with MSRA **(** Figure 1C3, Table S5).

About, 15.5% (17/110) and 13% (14/110) of the CACX samples showed methylation exclusively for *ROBO1* and *ROBO2* respectively, indicating that none of the events were epiphenomena (Table S2B). However, significant association was also found between the methylation statuses of *ROBO1* and *ROBO2* in both CIN and CACX (Table S3A), suggesting their possible cooperativity during cervical carcinogenesis.

**Figure 2:**
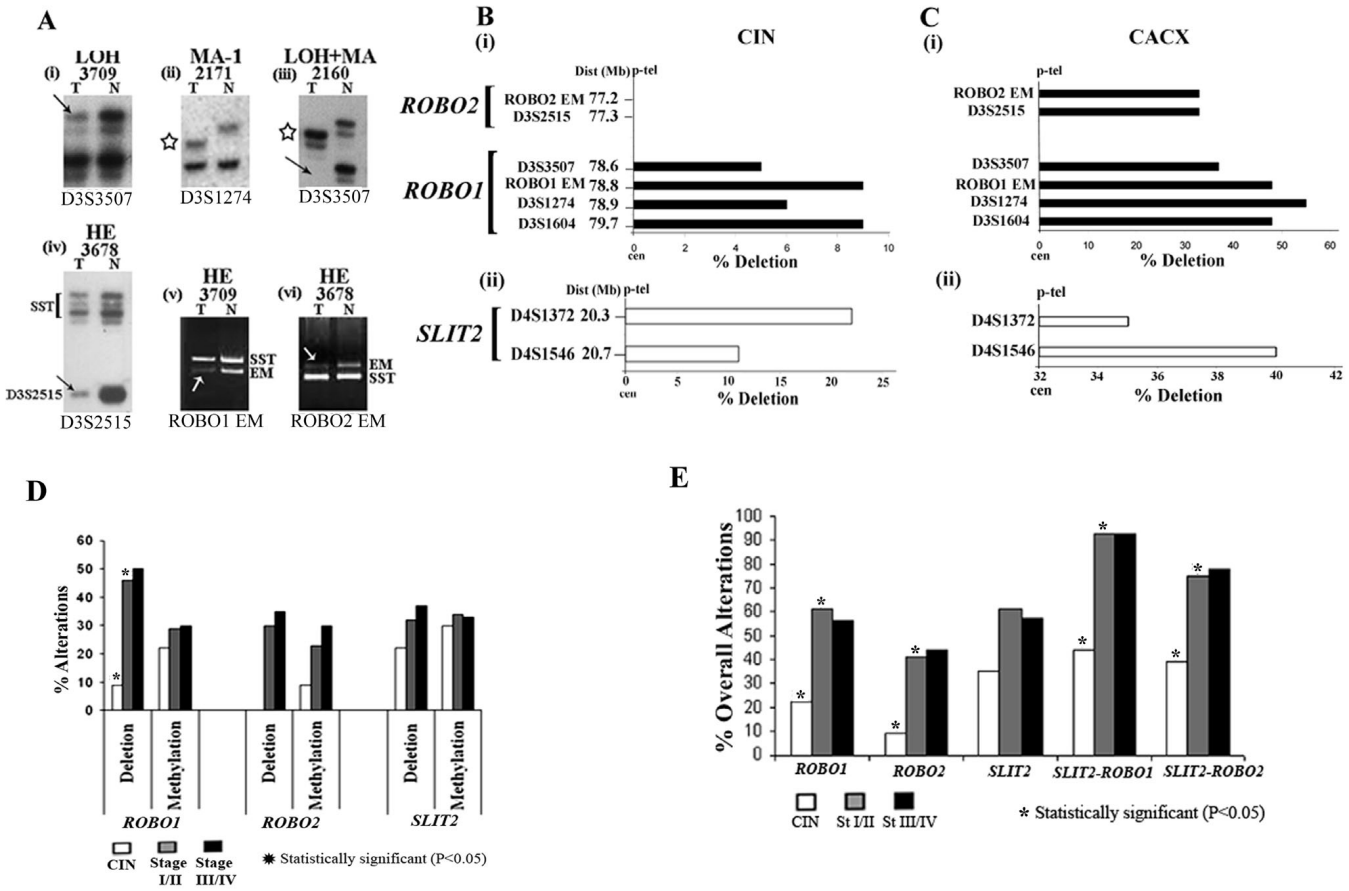
Representative autoradiographs showing deletion and microsatellite size alteration (MA) of cervical lesions, at different marker loci. (i) LOH: loss of heterozygosity, (ii) MA-1: microsatellite size alteration of one allele. (iii) LOH + MA: loss of one allele and microsatellite size alteration of the other. (iv) Hemizygous (HE) deletion of *ROBO2* locus as shown by D3S2515. (v) & (vi) HE deletion as shown by exonic markers (EM) from *ROBO1* and *ROBO2* respectively, *SST* used as control. The sample numbers and marker loci are indicated above and below the figure respectively. →: allelic loss, “*”: allelic size alteration. Deletion of *ROBO1/2* and *SLIT2* analyzed by microsatellite and exonic markers in (**B**) CIN and (**C**) CACX. T: Tumor DNA, N: DNA from normal cervix/PBL. (**D**) Pattern of deletion and methylation of *ROBO1/2* and *SLIT2* during disease progression. Asterisk denotes statistical significance (P<0.05). (**E**) Overall alteration patterns of the individual genes, *SLIT2-ROBO1* and *SLIT2-ROBO2* ligand-receptor pairs, during disease progression. Asterisk denotes statistical significance (P<0.05).

In case of *SLIT2* and *ROBO1* methylation was frequent (20–30%) in premalignant CIN lesions and remained comparable thereafter (Figure 2D). However, the methylation frequency of *ROBO2* increased gradually from CIN to subsequent stages of tumorigenesis, indicating that methylation was a late event for *ROBO2* inactivation (Figure 2D). Thus, promoter methylation of *SLIT2* and *ROBO1/2* might account for the lowered expression of these genes during cervical carcinogenesis.

### Validation of Promoter Methylation of the Genes by 5-Aza Deoxycytidine

To confirm if downregulation of the *SLIT2, ROBO1/2* and ncRNAs were due to methylation, demethylation experiment was performed by treatment of SiHa and HeLa cells with 5-Aza Deoxycytidine **(**5-aza dC). Dose dependent reactivation of *SLIT2* and *ROBO1/2* expression was observed in the cell lines w.r.t untreated controls. At the high 20 µM 5-aza dC concentration increase in expression frequencies were as follows: **SiHa**: SLIT2 (14.5) > ROBO2 (2.6) > ROBO1 (2.1) > BC043430 (1.8) > BC017743 (1.7) and **HeLa**: ROBO2 (4.6) > BC043430 (3) > SLIT2 (2.8) > BC017743 (2.5) > ROBO1 (1.9) (Figure 1D–F). The comparatively high expression of *ROBO2* in both cell lines and *SLIT2* in SiHa were in concordance with their methylation statuses. Differential increase in expression of ROBO1 and ncRNAs in both the cell lines, re-affirms transcription of these genes from different promoters. Thus, our data validates promoter methylation as one of the inactivating mechanisms of *SLIT2* and *ROBO1/2* in CACX development.

### Frequent Deletion of *SLIT2* and *ROBO1/2* loci

Genetic mechanisms like deletion, often results in inactivation of the candidate TSGs. In CIN lesions, deletion frequency of *SLIT2* was high (22%, 5/23) followed by *ROBO1* (9%, 2/23). However, *ROBO2* showed no deletion (Figure 2B, Table S2A,C). In CACX, *ROBO1* was highly deleted (48%, 53/110) followed by *SLIT2* (35%, 38/110) and *ROBO2* (33%, 36/110) (Figure 2C, Table S2B,D). None of these loci showed deletion in either SiHa or HeLa cells.

In CACX, deletion of *ROBO2* showed significant association with *ROBO1* and *SLIT2* (Table S3B), indicating their correlation in this carcinogenesis. The deletion of *ROBO2* is not an epiphenomenon of deletion of *ROBO1* as 11% (12/110) of the CACX samples showed deletion only of *ROBO2* (Table S2B). Microsatellite size alterations (MA) of *ROBO1* and *SLIT2* were infrequent in cervical tumors (Table S2A–D), unlike HNSCC (15). No homozygous deletion but other types of biallelic alterations [LOH+MA, MAII] was evident for *SLIT2* and *ROBO1* loci (Table S2A–D). The deletion frequency of *SLIT2* increased gradually during tumor progression (Figure 2D). However, in case of *ROBO1* there was significant increase in deletion frequency from CIN to stage I/II tumors and for *ROBO2*, frequency of deletion was high in stage I/II tumors and remained comparable in the subsequent stage (Figure 2D). These facts indicate that deletion was a late event during inactivation of the receptor genes and contributed towards inactivation of both *SLIT2* and *ROBO1/2* in CACX.

### Overall Alterations of *SLIT2* and *ROBO1/2*

Majority of the tumors (86%; 74/110) showed (epi) genetic alterations in at least one of the *SLIT2* and/or *ROBO1/2* genes, indicating their importance in cervical tumorigenesis. The overall alterations (deletion/methylation) of these genes in CIN and CACX were in the following order: **CIN**: *SLIT2* (35%; 8/23) > *ROBO1* (22%; 5/23) > *ROBO2* (9%; 2/23) (Figure 2E, Table S2A,C); **CACX**: *SLIT2* (59%; 65/110) > *ROBO1* (58%; 64/110) > *ROBO2* (43%; 47/110) (Table S2B,D).

Significant association between deletion and methylation of *SLIT2* and *ROBO1* in CIN, *ROBO1* and *ROBO2* in CACX and marginal significance of *SLIT2* in CACX were in concordance with Knudson’s modified two-hit hypothesis for candidate TSGs (Table S4).

The alterations of *SLIT2* were frequent (35%) in CIN lesions and increased gradually during subsequent stages of tumorigenesis (Figure 2E). However, for both *ROBO1* and *ROBO2*, alteration frequencies increased significantly from CIN to stage I/II tumors and remained comparable thereafter (Figure 2E), indicating that alterations of the receptors were late event during cervical carcinogenesis.

Overall alterations of *ROBO1* and *ROBO2* showed significant association in CIN and CACX, whereas *SLIT2* and *ROBO1* in CACX, suggesting their functional cooperativity in regulating the signaling pathways (Table S3C). Frequent alterations of *SLIT2* and/or *ROBO1* were seen in CIN (44%; 10/23) followed by significant increase in stage I/II (93%; 52/56) and stage III/IV tumors (93%; 50/54) (Figure 2E). Similar was the trend for *SLIT2* and/or *ROBO2* alterations (Figure 2E). This suggests that deregulation of these ligand-receptor interactions might have important role in the development of both premalignant cervical lesions and early invasiveness of the disease.

### Analysis of SLIT2 and ROBO1/2 Protein Expression

**Figure 3:**
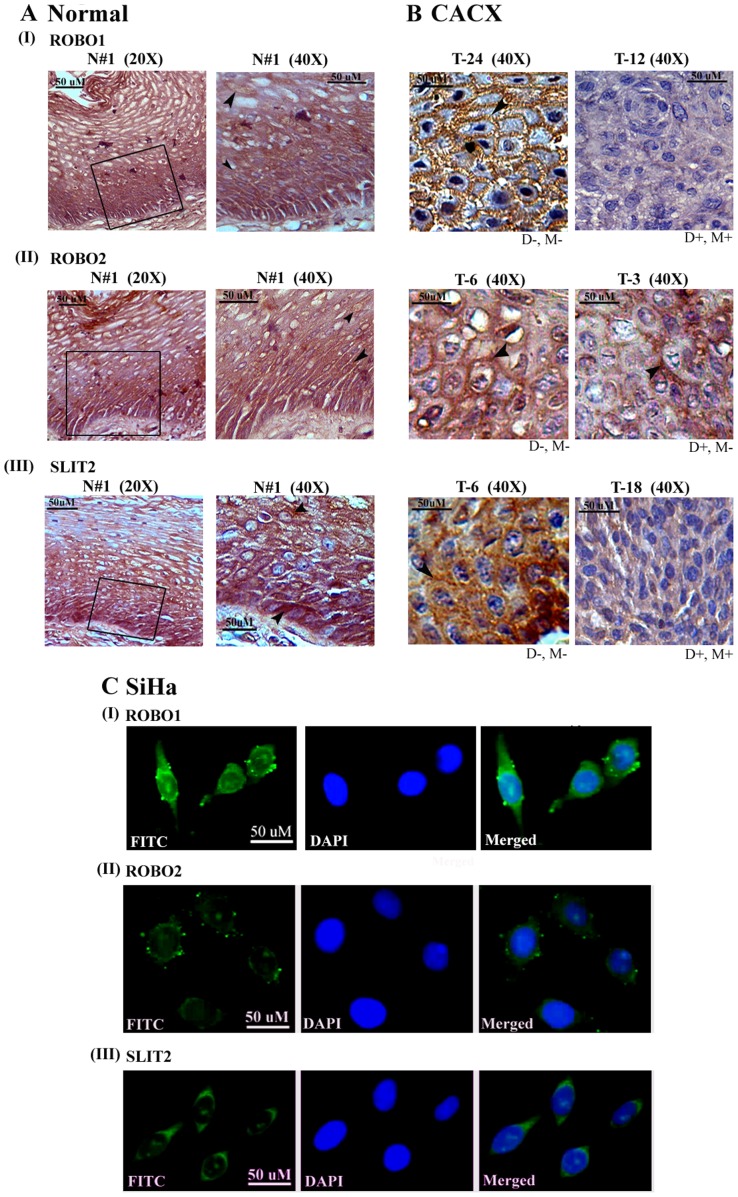
Immunohistochemical staining patterns of ROBO1, ROBO2 and SLIT2. (**A**) In normal cervical epithelium the basal and parabasal layer stained intensely for all the three proteins, whereas the intensity and frequency of stained cells reduced with further differentiation in the spinous layer. (**B**) In primary CACX expression pattern of these proteins were concordant with respective molecular alterations. (**C**) In SiHa cells ROBO1 and ROBO2 were membrane localized, whereas SLIT2 was present mostly in the cytoplasm. T: primary CACX sample; scale bars for both 20X and 40X is 50 µm; original magnifications are indicated in parenthesis. Magnification of panel **C** is 40X.

The association of alterations (deletion/methylation) and RNA expressions of *SLIT2* and *ROBO1/2* with their protein expressions was studied in primary CACX and the cell lines: SiHa and HeLa. In IHC analysis, expression of ROBO1/2 and SLIT2 were observed in the membrane and cytoplasm of the basal and parabasal layers of normal cervical epithelium and comparatively low expression in the spinous layer (Figure 3AI–III). The primary tumors without alterations (deletion/methylation) of these genes showed expression of these proteins similar to the basal/parabasal layers. However, significant association was seen between reduced/no expression of the proteins with their molecular alterations (Figure 3BI–III, Table 2).

**Table 2:** Correlation between deletion/methylation and reduced expression (RNA/protein) of *ROBO1/2* and *SLIT2* in CIN/CACX.

Sample	*ROBO1*			*ROBO2*			*SLIT2*		
	Del/Meth	mRNA	Protein	Del/Meth	mRNA	Protein	Del/Meth	mRNA	Protein
T1	D+	↓	nd	D−, M−	−	nd	D+	↓	nd
T2	M+	↓	Intermediate	M+	↓	Intermediate	D+, M+	↓	Absent
T3	D+	↓	Low	D+	↓	Low	D+, M+	↓	Absent
T4	D+, M+	↓	nd	D−, M−	↑	nd	D−, M−	−	nd
T5	D+, M+	↓	nd	D+, M+	↓	nd	D+, M+	↓	nd
T6	D+	↓	Low	D−, M−	−	Intermediate	D−, M−	−	Intermediate
T7	M+	↓	nd	D+, M+	↓	nd	D+	↓	nd
T8	D+	↓	Low	D+	↓	Absent	D+	↓	Absent
T9	D−, M−	↓	nd	D+, M+	↓	nd	D+, M+	↓	nd
T10	D+	↓	Low	D−, M−	↓	Intermediate	D−, M−	↓	Intermediate
T11	D+	↓	nd	D+	↓	nd	D−, M−	↓	nd
T12	D+, M+	↓	Absent	D+	↓	Low	D+	↓	Low
T13	M+	↓	nd	M+	↓	nd	D−, M−	↓	nd
T14	D−, M−	↓	nd	D+	↓	nd	D+	↓	nd
T15	D+, M+	↓	Absent	D+, M+	↓	Absent	D+, M+	↓	Absent
T16	D+, M+	↓	nd	M+	↓	nd	M+	↓	nd
T17	D+	↓	nd	D−, M−	−	nd	D−, M−	−	nd
T18	D+, M+	↓	Low	D+, M+	↓	Absent	D+, M+	↓	Low
T19	D−, M−	↓	nd	D−, M−	−	nd	D−, M−	−	nd
T20	D+, M+	↓	nd	D−, M−	↑	nd	D−, M−	−	nd
T21	D+	↓	Low	M+	↓	Intermediate	D−, M−	↓	Intermediate
C1	D−, M−	ND	Intermediate	D−, M−	ND	Intermediate	D+, M+	ND	Low
C2	D−, M−	ND	Intermediate	D−, M−	ND	Intermediate	D+, M+	ND	Low
C3	D−, M−	ND	Intermediate	D−, M−	ND	Intermediate	D−, M−	ND	Intermediate
T-24	D−, M−	ND	Intermediate	D+	ND	Low	D+	ND	Low
T-25	D+	ND	Low	D−, M−	ND	Intermediate	D−, M−	ND	Intermediate
T-26	D+, M+	ND	Low	D+, M+	ND	Low	D−, M−	ND	Intermediate
***P*** **-value**	Non-evaluable			0.00016*			0.003*		

Samples C1, C2 and C3 are CIN lesions.

D+/−, Deletion (HE, HM, LOH) positive/negative; M+/−, methylation positive/negative; Del/Meth: Deletion or methylation. ↓: Reduced mRNA expression (≥2 folds); ↑: Increased mRNA expression (≥2 folds); -: Reduced/Increased mRNA expression (<2 folds). nd, not done due to insufficient/scanty paraffin embedded tumor tissue; ND, Fresh tissues unavailable for RNA isolation. *, statistically significant (*P*<0.05).

SLIT2 and ROBO2 expressions were reduced in SiHa and HeLa cells. Expressions of ROBO1 and ROBO2 were observed with some discrete foci on the membrane of SiHa cells (Figure 3CI–III). Similar localizations of these proteins were seen in HeLa, without any focus formation (Figure S1). This might be due to some modifications of these receptors in SiHa, leading to their aggregation on the membrane and subsequent dysfunction of the downstream pathway(s). Cytoplasmic expression of SLIT2 was observed in SiHa cells, in concordance with the reports of Singh et al [2007] (12), in HeLa. In primary tumors, reduced/no expression of SLIT2 and/or ROBO1 was found in 93% (14/15) samples and of SLIT2 and/or ROBO2 in 67% (10/15) (Table 2), indicating the deregulation of these ligand-receptor(s) interactions as critical events in cervical carcinogenesis.

### Clinico-pathological Association of *SLIT2* and *ROBO1/2*

The alterations of *SLIT2* and *ROBO1/2* were correlated with the clinico-pathological/survival parameters in order to uncover their prognostic significance (if any) for early diagnosis and prediction of patient outcome. HPV, an important causative agent of CACX, was detected in 86.5% (115/133) of the cervical lesions. Of these, 88% (101/115) were HPV16 positive and 12% (14/115) were HPV18 positive. HPV infection was not significantly associated with tumor stage, grade, nodal status, parity, age at sexual debut (Table 1) and *SLIT2-ROBO1/2* alterations (data not shown). Similarly, chi-square analysis revealed significant association of *SLIT2-ROBO1/2* alterations with tumor stage, however no such associations were observed with the other clinico-pathological parameters (grade, nodal status, parity etc; data not shown).

**Figure 4:**
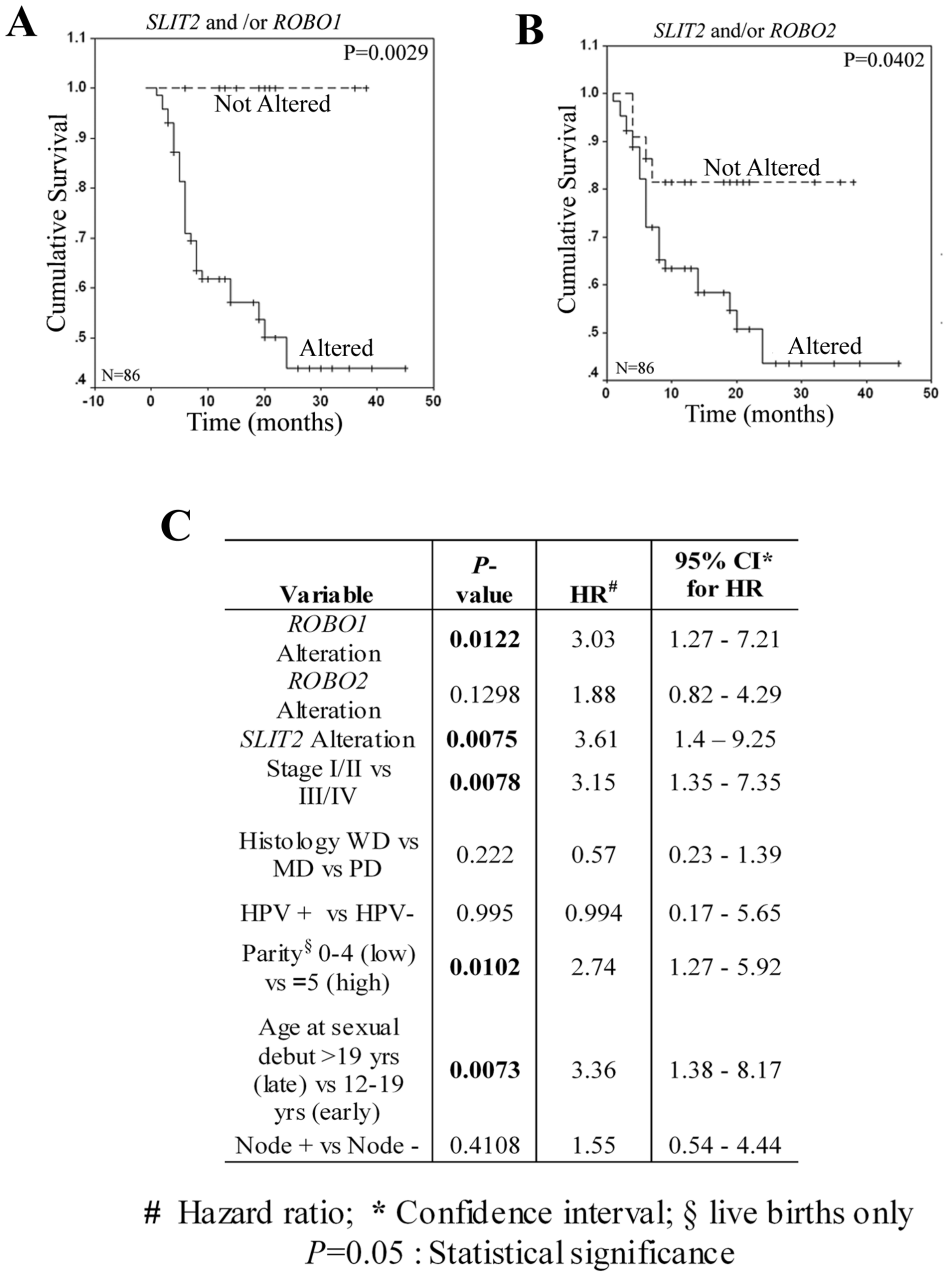
Kaplan-Meier analysis of survival (up to 5 years) of CACX patients. Alterations of (**A**) *SLIT2* and/or *ROBO1* and (**B**) *SLIT2* and/or *ROBO2* ligand-receptor pairs were significantly associated with poor patient outcome [OS]. (**C**) Representation of Cox Multivariate analyses of genetic, clinical and etiological parameters in predicting outcome of CACX patients. N: total number of samples and P<0.05 denotes statistical significance.

The Kaplan-Meier (K–M) survival analysis revealed significantly reduced overall survival (OS) of CACX patients with alterations of *SLIT2*, *ROBO1* and *ROBO2* (Figure S2A–C), indicating their prognostic significances. Interestingly, alterations of *SLIT2* and/or *ROBO1* and *SLIT2* and/or *ROBO2* predicted poor OS of the patients, suggesting abrogation of these ligand-receptor interactions were directly equated to poor prognosis (Figure 4A,B). The Cox multivariate analysis indicated that alterations of *SLIT2* and *ROBO1* along with advanced tumor stage (III/IV), multiparity (≥5) and early sexual debut (<19 years) were determinants of poor prognosis for CACX patients (Figure 4C), thereby enabling efficient classification of the high-risk patients.

## Discussion

The aim of this study was to investigate the association of ligand-receptor genes *SLIT2* and *ROBO1/2* in cervical carcinogenesis. To this end, at first, expression (RNA) profiles of these genes were analyzed in primary CACX samples and CACX cell lines. Highly reduced expressions of these genes were revealed. Then alterations (deletion/methylation) of the genes were analyzed to understand the mechanism of reduced expression. Alterations of the genes were then correlated with their expression (RNA and protein) profile. In addition molecular alterations of the genes were correlated with different clinico-pathological parameters. Reduced RNA and protein expression of *SLIT2* and *ROBO1/2* were observed in primary CACX, in concordance with the previous reports [12], [13]. However, none prior to us reported the expression pattern of SLIT2, ROBO1 and ROBO2 concomitantly in primary CACX. Alike us reduced expression of these genes were also reported in a plethora of other malignancies including HNSCC, gliomas and carcinomas of liver, lung, breast, kidney etc [15], [27]–[33]. However, overexpression of these genes were also reported in prostate and breast cancers [34], [35]. In addition, Angeloni et al., (2006) [16] suggested that expression of *ROBO1* could be regulated by two ncRNAs encoded by the intron2 of *ROBO1*. However, our data suggested that there was no concordance between the expression profiles of *ROBO1* and the ncRNAs, as observed in HNSCC [15].

Overall alterations of atleast one member of the *SLIT2-ROBO1/2* cascade were observed in 43% CIN lesions and 95% CACX, indicating the importance of deregulation of this pathway for cervical carcinogenesis. Alterations of *SLIT2* was observed in 35% CIN lesions with the frequency increasing gradually in subsequent stages, indicating this as an early event, in concordance with the reports of Narayan et al., (2006) [13]. In addition deletion or methylation mediated deregulation of *SLIT2* has already been reported in various malignancies including gliomas, neuroblastoma, Wilm’s tumor, leukemia and carcinomas of lung, breast, kidney, colon etc though, none reported the stage wise correlation pattern [15], [28]–[30], [33], [36]–[40]. The alteration frequencies of *ROBO1* and *ROBO2* increased significantly from CIN to stage I/II tumors, indicating that these receptors were inactivated during the development of early invasive cervical tumors. Alike *SLIT2*, deletion or methylation mediated inactivation of *ROBO1* have also been reported in multiple malignancies including lung, breast, HNSCC etc [15], [27], [41]. However, alterations of *ROBO2* have been reported only in HNSCC, as yet [15].

In premalignant CIN lesions, alterations of *SLIT2* and/or *ROBO1* and *SLIT2* and/or *ROBO2* ligand-receptor pairs were observed in 44% and 39% cases respectively, which increased significantly in the subsequent stage I/II tumors and remained comparable thereafter. This fact corroborates that abrogation of the ligand-receptor interaction was necessary for initiation and progression of CACX. As yet none has comprehensively reported the importance of loss of these ligand-receptor interactions in cervical carcinogenesis of Indian patients.

A close scrutiny of the methylation and deletion frequencies of *SLIT2* and *ROBO1* during progression of CACX, indicated that methylation was the more frequent alteration that occurred early during carcinogenic progression. Although, in case of *ROBO2*, promoter methylation was the late event nonetheless, it was predominant over deletion. Predominant methylation and infrequent deletion of *SLIT2* (64%, 9%) and *ROBO1* (46%, 10%) were also reported in CACX of Western patients [13]. The deletion analysis was done in a cohort of 30 tumors compared to 133 in our analysis, thus accounting for the difference in results between the two studies [42]. Similarly the methylation frequency of *SLIT2* in our study was 34% and differed widely from that reported by Narayan et al., (2006) [13], possibly due to age, ethnicity and lifestyle variation of the patients included in both the studies [42]–[44].

The interaction between *SLIT2* and *ROBO1* triggers a number of downstream pathways, controlling diverse cellular processes namely; cell proliferation, apoptosis, motility etc [14]. The most prominent effectors includes srGAP, CDC42, β-catenin, Netrin-1, PI3K, CXCR4 etc [14], [45]. Detailed study of each of these pathways is warranted for identification of diagnostic or therapeutic targets of CACX, from among the effector molecules of the *SLIT2-ROBO1* signaling pathway.

In this study molecular alterations of the individual genes, *SLIT2* and/or *ROBO1* and *SLIT2* and/or *ROBO2* pairs showed differential association with the disease progression and predicted poor patient outcome. Moreover Cox multivariate analysis revealed that alterations of *SLIT2* and *ROBO1* coupled with advanced tumor stage (III/IV), multiparity (≥5) and early sexual debut (<19 years) were predictors of poor prognosis for CACX patients.

Thus we can conclude that abrogation of *SLIT2-ROBO1* and *SLIT2-ROBO2* signaling pathways are necessary for the initiation and progression of CACX. Since these ligand-receptor genes possess prognostic implications, they could be utilized as early diagnostic or prognostic markers for CACX.

## Supporting Information

Figure S1 **Immunofluorescence analysis of ROBO1, ROBO2 and SLIT2 in HeLa cells.** **(A–B)** Membrane localization of ROBO1/2 and **(C)** cytoplasmic/membrane localization of SLIT2. Scale bars are 50 µm, magnifications: 40X.
(TIF)

Figure S2 **Kaplan-Meier survival analysis (up to 5 years) of CACX patients.** **(A–C)** Alteration of *ROBO1*, *ROBO2* and *SLIT2* significantly associated with poor overall survival. **(D)** Alteration of atleast one of the ligand or receptors predicted poor patient outcome. N: total number of samples.
(TIF)

Table S1 **List of oligonucleotide primers.**
(XLS)

Table S2 **Allelic alterations of** ***ROBO1*** **,** ***ROBO2*** **and** ***SLIT2*** **in primary cervical lesions.**
(XLS)

Table S3 **Associations between methylation, deletion and overall alterations of** ***SLIT2*** **-** ***ROBO1/2*** **in the CIN and CACX samples.**
(DOC)

Table S4 **Correlation between deletion and methylation of** ***ROBO1/2*** **and** ***SLIT2*** **in CIN/CACX samples.**
(DOC)

Table S5 **Correlation between MSRA and MSP analyses.**
(DOC)

Information S1 **Supplementary Materials and Methods.**
(DOC)
