# Supplementary figures and images for: Inactivation of SLIT2-ROBO1/2 Pathway in Premalignant Lesions of Uterine Cervix: Clinical and Prognostic Significances
Source: PLoS One. 2012 Jun 13;7(6):e38342. doi: 10.1371/journal.pone.0038342 (PMC3374764; doi:10.1371/journal.pone.0038342)

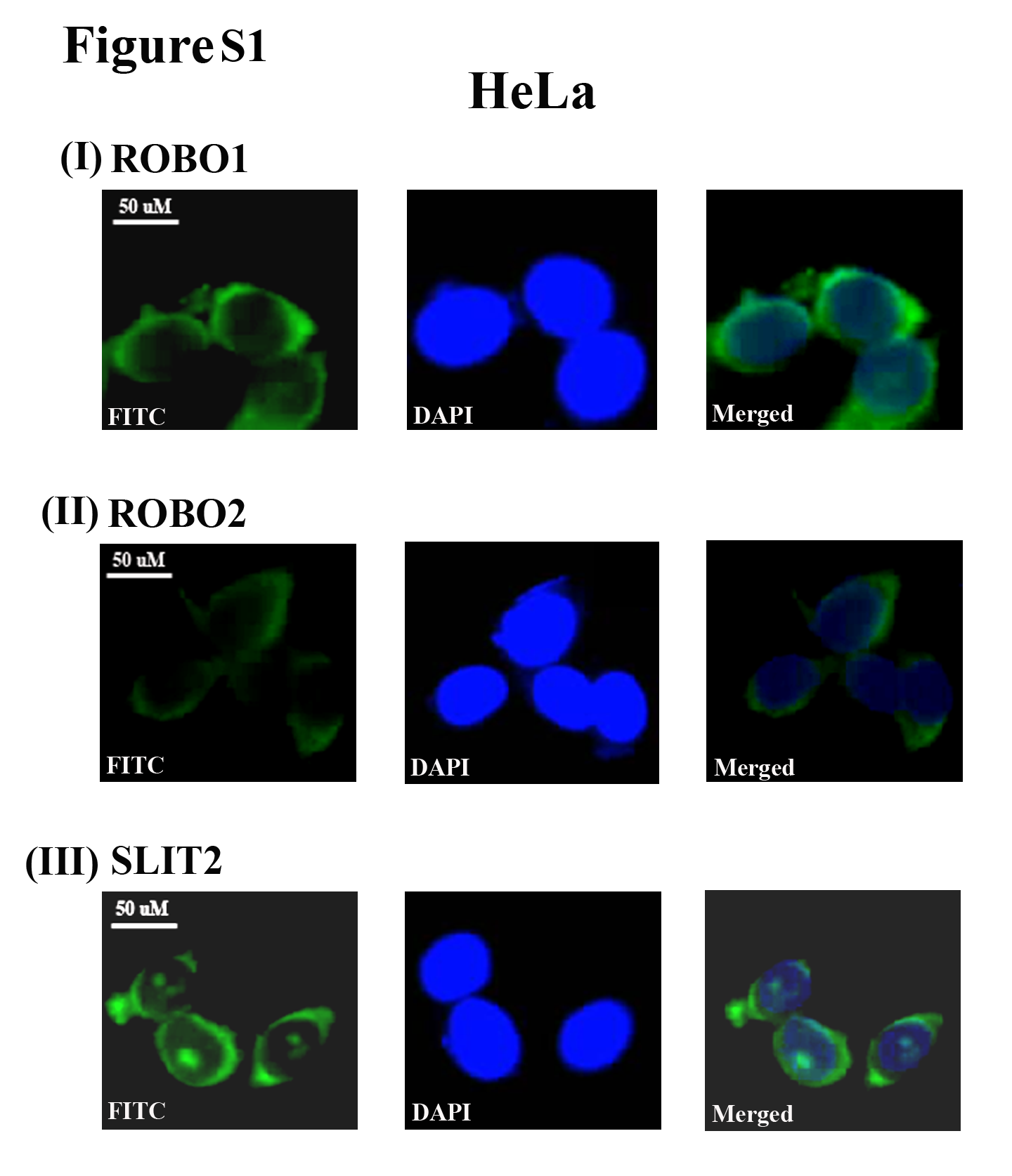

Supplement: Figure S1 — Immunofluorescence analysis of ROBO1, ROBO2 and SLIT2 in HeLa cells. (A–B) Membrane localization of ROBO1/2 and (C) cytoplasmic/membrane localization of SLIT2. Scale bars are 50 µm, magnifications: 40X. (TIF) [file pone.0038342.s001.tif]

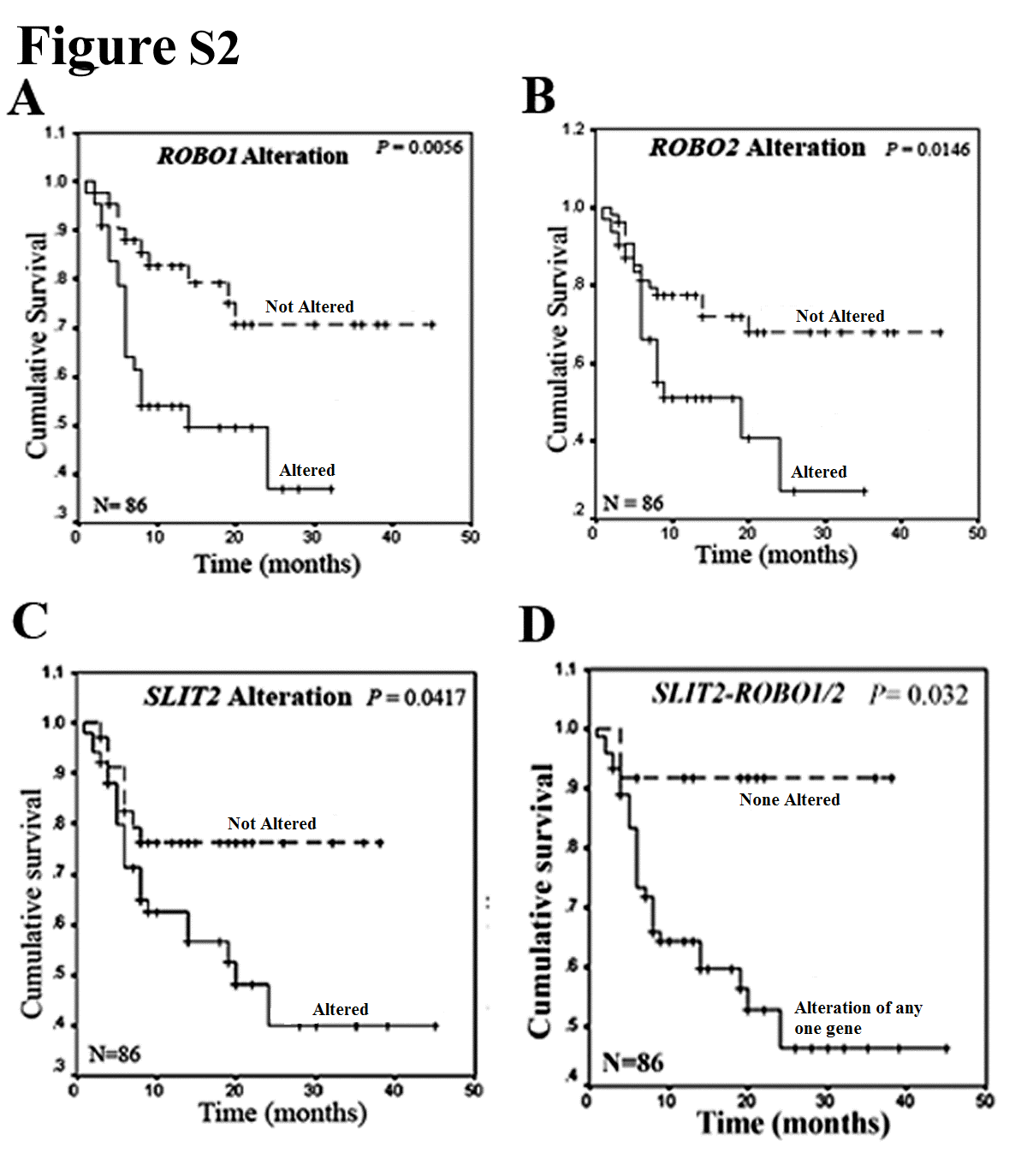

Supplement: Figure S2 — Kaplan-Meier survival analysis (up to 5 years) of CACX patients. (A–C) Alteration of ROBO1, ROBO2 and SLIT2 significantly associated with poor overall survival. (D) Alteration of atleast one of the ligand or receptors predicted poor patient outcome. N: total number of samples. (TIF) [file pone.0038342.s002.tif]
