# Supplementary material for: Inactivation of SLIT2-ROBO1/2 Pathway in Premalignant Lesions of Uterine Cervix: Clinical and Prognostic Significances
Source: PLoS One. 2012 Jun 13;7(6):e38342. doi: 10.1371/journal.pone.0038342 (PMC3374764; doi:10.1371/journal.pone.0038342)
Supplement: Table S3 — Associations between methylation, deletion and overall alterations of SLIT2 - ROBO1/2 in the CIN and CACX samples. (DOC) [file pone.0038342.s005.doc]

**Table S3A** Association between methylation of *SLIT2*-*ROBO1/2* in the CIN and CACX samples

|  | | **CIN^#^** | | | | | | **CACX^§^** | | | | | |
| --- | --- | --- | --- | --- | --- | --- | --- | --- | --- | --- | --- | --- | --- |
|  |  | ***ROBO1*** | | ***ROBO2*** | | ***SLIT2*** | | ***ROBO1*** | | ***ROBO2*** | | ***SLIT2*** | |
|  |  | **M+** | **M-** | **M+** | **M-** | **M+** | **M-** | **M+** | **M-** | **M+** | **M-** | **M+** | **M-** |
| ***ROBO1*** | **M+** | - | - | 2 | 3 | 2 | 3 | - | - | 14 | 18 | 7 | 25 |
|  | **M-** |  |  | 0 | 18 | 5 | 13 |  |  | 15 | 63 | 30 | 47 |
| ***P* value** | | - | | **0.0049** | | 0.599 | | - | | **0.008** | | 0.086 | |
| ***ROBO2*** | **M+** | - | - | - | - | 0 | 2 | - | - | - | - | 8 | 21 |
|  | **M-** |  |  |  |  | 7 | 14 |  |  |  |  | 30 | 52 |
| ***P^*^* value** | | - | | - | | 0.327 | | - | | - | | 0.38 | |

**Table S3B** Association between deletion of *SLIT2*-*ROBO1/2* in the CIN and CACX samples

|  | | **CIN^#^** | | | | | | **CACX^§^** | | | | | |
| --- | --- | --- | --- | --- | --- | --- | --- | --- | --- | --- | --- | --- | --- |
|  |  | ***ROBO1*** | | ***ROBO2*** | | ***SLIT2*** | | ***ROBO1*** | | ***ROBO2*** | | ***SLIT2*** | |
|  |  | **D+** | **D-** | **D+** | **D-** | **D+** | **D-** | **D+** | **D-** | **D+** | **D-** | **D+** | **D-** |
| ***ROBO1*** | **D+** | - | - | 0 | 2 | 1 | 1 | - | - | 25 | 28 | 22 | 31 |
|  | **D-** |  |  | 0 | 21 | 4 | 17 |  |  | 11 | 46 | 16 | 41 |
| ***P* value** | | - | | Non-evaluable | | 0.31 | | - | | **0.0018** | | 0.1385 | |
| ***ROBO2*** | **D+** | - | - | - | - | 0 | 0 | - | - | - | - | 21 | 16 |
|  | **D-** |  |  |  |  | 5 | 18 |  |  |  |  | 18 | 56 |
| ***P^*^* value** | | - | | - | | Non-evaluable | | - | | - | | **0.00074** | |

**Table S3C** Association between alterations of *SLIT2*-*ROBO1/2* in the CIN and CACX samples

|  | | **CIN^#^** | | | | | | **CACX^§^** | | | | | |
| --- | --- | --- | --- | --- | --- | --- | --- | --- | --- | --- | --- | --- | --- |
|  |  | ***ROBO1*** | | ***ROBO2*** | | ***SLIT2*** | | ***ROBO1*** | | ***ROBO2*** | | ***SLIT2*** | |
|  |  | **A+** | **A-** | **A+** | **A-** | **A+** | **A-** | **A+** | **A-** | **A+** | **A-** | **A+** | **A-** |
| ***ROBO1*** | **A+** | - | - | 2 | 3 | 3 | 2 | - | - | 33 | 31 | 27 | 37 |
|  | **A-** |  |  | 0 | 18 | 5 | 13 |  |  | 14 | 32 | 38 | 8 |
| ***P* value** | | - | | **0.0049** | | 0.18 | | - | | **0.027** | | **0.00002** | |
| ***ROBO2*** | **A+** | - | - | - | - | 1 | 1 | - | - | - | - | 28 | 19 |
|  | **A-** |  |  |  |  | 7 | 14 |  |  |  |  | 37 | 26 |
| ***P** value** | | - | | - | | 0.6363 | | - | | - | | 0.929 | |

**#** Cervical intraepithelial neoplasia, **§** Uterine cervical carcinoma, D+/M+ Deletion/Methylation positive; D-/M- Deletion/Methylation negative, A+/A- Alteration positive/negative, ******* Statistically significant (*P*<0.05).
