# Supplementary material for: Inactivation of SLIT2-ROBO1/2 Pathway in Premalignant Lesions of Uterine Cervix: Clinical and Prognostic Significances
Source: PLoS One. 2012 Jun 13;7(6):e38342. doi: 10.1371/journal.pone.0038342 (PMC3374764; doi:10.1371/journal.pone.0038342)
Supplement: Table S4 — Correlation between deletion and methylation of ROBO1/2 and SLIT2 in CIN/CACX samples. (DOC) [file pone.0038342.s006.doc]

**Table S4** Correlation between deletion and methylation of *ROBO1/2* and *SLIT2* in CIN/CACX samples

|  |  | ***ROBO1*** | | | |  | ***ROBO2*** | | | |  | ***SLIT2*** | | | |
| --- | --- | --- | --- | --- | --- | --- | --- | --- | --- | --- | --- | --- | --- | --- | --- |
|  |  | **Methylation** | | | |  | **Methylation** | | | |  | **Methylation** | | | |
|  |  | **CIN** | | **CACX** | |  | **CIN** | | **CACX** | |  | **CIN** | | **CACX** | |
|  |  | M+ | M- | M+ | M- |  | M+ | M- | M+ | M- |  | M+ | M- | M+ | M- |
| **Deletion** | D+ | 2 | 0 | 20 | 33 |  | 0 | 0 | 18 | 18 |  | 4 | 1 | 9 | 29 |
|  | D- | 3 | 18 | 12 | 45 |  | 2 | 21 | 11 | 63 |  | 3 | 15 | 28 | 42 |
|  | P-value | 0.0049* | | 0.054 | |  | Non-evaluable | | 0.000087* | |  | 0.0065* | | 0.0879 | |
|  |  |  |  |  |  |  |  |  |  |  |  |  |  |  |  |

Asterisk represents statistical significance (*P*≤0.05). *P*-values ranging between 0.051-0.09 were considered marginally significant.
