# Supplementary material for: Inactivation of SLIT2-ROBO1/2 Pathway in Premalignant Lesions of Uterine Cervix: Clinical and Prognostic Significances
Source: PLoS One. 2012 Jun 13;7(6):e38342. doi: 10.1371/journal.pone.0038342 (PMC3374764; doi:10.1371/journal.pone.0038342)
Supplement: Table S5 — Correlation between MSRA and MSP analyses. (DOC) [file pone.0038342.s007.doc]

**Table S5.** Correlation between MSRA and MSP analyses

| Samples | Methylation status of *ROBO1* | | Methylation status of *ROBO2* | | Methylation status of *SLIT2* | |
| --- | --- | --- | --- | --- | --- | --- |
|  | **MSRA** | **MSP** | **MSRA** | **MSP** | **MSRA** | **MSP** |
| 3385 | - | - | - | - | - | - |
| 5 | + | + | + | + | - | - |
| 1319 | - | - | - | - | - | + |
| 5246 | + | + | - | + | - | - |
| 3112 | - | - | - | - | - | - |
| 4025 | - | - | - | - | + | + |
| 3237 | - | - | - | - | + | + |
| 3229 | + | + | + | + | - | - |
| 1641 | - | - | - | - | + | + |
| 6253 | - | + | + | + | - | - |
| 165 | - | - | + | + | - | - |
| 3920 | + | + | + | + | - | - |
| 2986 | - | - | - | - | - | + |
| 1435 | + | + | + | + | - | - |
| 4483 | + | + | + | + | - | - |
| T3 | - | - | - | - | + | + |
| T7 | + | + | + | + | - | - |
| T10 | - | - | - | - | + | + |
| T13 | - | - | + | + | - | - |
| T17 | - | + | - | - | - | - |
| **% methylation** | 35 (7/20) | 45 (9/20) | 45 (9/20) | 50 (10/20) | 25 (5/20) | 35 (7/20) |
| **P-value** | **0.00028*** | | **0.00005*** | | **0.00043*** | |

Asterisk denotes statistical significance (P-value <0.05)

+: Methylated CpG; -: Unmethylated CpG
