# Supplementary material for: Inactivation of SLIT2-ROBO1/2 Pathway in Premalignant Lesions of Uterine Cervix: Clinical and Prognostic Significances
Source: PLoS One. 2012 Jun 13;7(6):e38342. doi: 10.1371/journal.pone.0038342 (PMC3374764; doi:10.1371/journal.pone.0038342)
Supplement: Information S1 — Supplementary Materials and Methods. (DOC) [file pone.0038342.s008.doc]

**Supplement 1: Supplementary Materials and Methods**

**RNA expression analysis**

Freshly collected tissue was taken in TRIzol reagent for total RNA isolation according to the manufacturer’s protocol. The cDNA was synthesized from 1μg total RNA using Random hexamer (Invitrogen, USA) and Superscript III (Invitrogen, USA). Real time quantitation of the candidate genes was performed using SYBR-green PCR assay and β2-microglobulin as control. Briefly 2μl of c-DNA was amplified in 25 μl reaction volume containing 12.5 μl of 2XSYBR-green master mix (Applied Biosystems, USA) and 1 μl of each primer. Each sample was loaded in triplicate and run for 40 cycles on an ABI prism 7500 machine (Applied Biosystems, USA). After each run melting curves was analyzed to confirm amplification of specific transcripts. The relative level of gene expression was determined by comparative threshold cycle (Ct) method [18, 19].

**Microdissection and DNA Extraction**

Cryosections (5μm) were made from frozen tissues, representative sections were stained with hematoxylin-eosin and then examined by two independent pathologists who marked the dysplastic epithelium (CIN) / tumor rich regions (CACX). The sections representative of the above stained and marked portion were microdissected [20] under dissecting microscope (Leica MZ 16, Germany) and from these dissected portions high molecular weight DNA was isolated using phenol-chloroform [20, 21].

**Methylation analysis**

Methylation sensitive restriction analysis (MSRA) utilized the ability of methylation-sensitive restriction enzymes viz: MspI/HpaII [Roche Diagnostics, Germany] [22] to cleave sites with unmodified cytosines and not those with 5-methylcytosines. About, 100 ng of genomic DNA samples were digested overnight with methyl-sensitive restriction enzyme *Hpa*II and its methylation in-sensitive isoschizomer *Msp*I separately; the digested DNA was PCR amplified, run on 2% agarose gel and scored for methylation positivity. Mock digestion was done with each sample devoid of enzyme. The 445-bp fragment of *β-3A adaptin* gene *(K1*) and 229-bp fragment of *RARβ2* exon-1 (*K2*) were used as digestion and integrity controls respectively [22].

The methylation data obtained by MSRA was validated by methylation-specific PCR (MSP) in 25 randomly selected primary cervical lesions after bisulfite modification of the DNA [23]. Briefly, 5μg of genomic DNA was denatured with 0.2M NaOH for 15min at 37°C. Cytosines were sulfonated in the presence of 3.0 M sodium-bisulfite (Sigma) and 10mM hydroquinone (Sigma) for 16hr at 50°C. Thereafter, the DNA samples were desalted using Wizard DNA Clean-Up System (Promega, Madison, WI, USA) and desulfonated in 0.3M NaOH at 37°C for 15 min. Finally, the treated DNA samples were precipitated with ethanol and resuspended in TE (pH 7.4). The PCR of the modified DNA was done with methylation specific/unmethylation specific (M/U) primers set (Table S1) according to the standard procedure [23]. PCR products were analyzed on 2% agarose gels, visualized under UV illumination and photographed.

# Deletion analysis

In microsatellite based deletion analysis, for informative markers, a standard polymerase chain reaction (PCR) containing [γ-p32] ATP end labeled forward primer was done in a 20μl reaction volume. PCR products were electrophoresed on 7% denaturing polyacrylamide sequencing gel containing 8M urea and autoradiographed. Loss of heterozygosity (LOH) was detected by densitometric scanning (Bio-Rad GS-800, USA) and the scoring of LOH and microsatellite size alterations (MA) was done as described previously [20]. LOH was scored if there was complete loss of one allele/50% reduction in band intensities of one allele in the tumor compared to the same allele in normal. The value was calculated as ratio of band intensities of the larger to the smaller alleles in tumor divided by the same ratio in the normal sample. A LOH index >1.5 (loss of smaller allele) or <0.67 (loss of larger allele). MA was detected as a shift in one (MA-I) or both (MA-II) alleles compared to their normal alleles. MA of one allele and loss of the other was denoted as LOH+MA. In case of non-informative microsatellite markers, deletion was analyzed by multiplex PCR, using [γ-P^32^] end labeled forward primer of non-informative and a control marker [SST (3q27.3) and D4S2376 (4q31.21)]. PCR products were run in 7% denaturing polyacrylamide sequencing gel containing 8M urea and autoradiographed [12]. For exonic markers (EM) multiplex PCR was done using the concerned EM along with a control (SST EM at 3q27.3). The products were electrophoresed in 2% agarose gel, stained with ethidium bromide, visualized and scanned in Gel Documentation System (Bio-Rad, USA). SST was used as control locus for the non-informative markers of chr 3 (D3S2515, D3S1604) and D4S2376 served as control locus for D4S1372.

The number of alleles at the locus of interest was evaluated from the signal intensities by densitometric scanning (Bio-Rad GS-800, USA). Deletion, amplification or retention of both alleles was calculated as follows [24].

LOI (T) / CL (T)

Allelic number = 2 x --------------------

LOI (N) / CL (N)

Where T= tumor DNA and N = normal DNA. LOI = Locus of Interest, CL= Control locus (SST & D4S2376). The allele values of <0.6, 0.9 - 1.3, >1.7 - < 5.0 and >5.0 were considered as: homozygous, hemizygous deletion, retention of both alleles and amplification of alleles respectively.

**Immunohistochemical analysis**

About 3-5 μm paraffin sections of normal cervical tissue and primary CACX samples (n=15) were dewaxed, rehydrated and reacted overnight with primary antibodies [Goat polyclonal IgG sc-16611, sc-16615 and sc-16619 for ROBO1, ROBO2 and SLIT2 respectively] Santa Cruz, CA, USA at a dilution of 1:100 at 4^o^C. Horseradish peroxidase (HRP)-conjugated rabbit anti-goat secondary antibody (sc-2768) Santa Cruz, CA, USA was added at 1:500 dilutions. The slides were developed using 3-3' diaminobenzidine (DAB) as the chromogen and counterstained with hematoxylin. The staining intensity (1 = weak, 2 = moderate, 3 = strong) and the percentage of positive cells (<1 = 0, 1-20 = 1, 20-50 =2, 50-80 = 3 and >80 =4) were detected by two observers independently and by combining the two scores, final evaluation of expression was done (0-2 = low, 3-5 = intermediate, 6-7 = high) [25].

SiHa and HeLa cells were cultured on coverslips till sub-confluency, fixed with methanol, blocked with BSA, incubated with the respective primary antibodies for ROBO1, ROBO2 and SLIT2 followed by fluorescein isothiocyanate (FITC)-conjugated rabbit anti-goat secondary antibody [sc-2777], washed and briefly incubated with DAPI (sc-3598) and subsequently mounted on slides, viewed and photographed by fluorescence microscope (Leica DM 4000B; Germany).
